# Solving Fuzzy Fractional Differential Equations Using Zadeh's Extension Principle

**DOI:** 10.1155/2013/454969

**Published:** 2013-09-04

**Authors:** M. Z. Ahmad, M. K. Hasan, S. Abbasbandy

**Affiliations:** ^1^Institute of Engineering Mathematics, Universiti Malaysia Perlis, Kampus Tetap Pauh Putra, 02600 Arau, Perlis, Malaysia; ^2^School of Information Technology, Faculty of Information Science and Technology, Universiti Kebangsaan Malaysia, 43600 Bangi, Selangor, Malaysia; ^3^Department of Mathematics, Imam Khomeini International University, Ghazvin 34149-16818, Iran

## Abstract

We study a fuzzy fractional differential equation (FFDE) and present its solution using Zadeh's extension principle. The proposed study extends the case of fuzzy differential equations of integer order. We also propose a numerical method to approximate the solution of FFDEs. To solve nonlinear problems, the proposed numerical method is then incorporated into an unconstrained optimisation technique. Several numerical examples are provided.

## 1. Introduction

Fractional calculus is an important branch in mathematical analysis. It is a generalisation of ordinary calculus that allows noninteger order. At the beginning, it was slowly established. However, after Leibniz and Newton invented differential calculus, it has been a subject of interest among mathematicians, physicists, and engineers. Consequently, the theory of fractional calculus has been extensively developed and influenced in many areas of discipline. The fractional integral and fractional derivative of Riemann-Liouville, for example, have been applied to solve many mathematical problems [1–6]. One of the particular interests is the case of solving fractional differential equations [7–9]. The fractional derivative of Riemann-Liouville, however, has a common characteristic. It requires a quantity of fractional derivative of unknown solution at initial point. In practice, we do not clearly know what the meaning of the fractional derivative at that point is. In other words, the required quantity cannot be measured and perhaps may not be available [10, 11].

The well-known and popularly used method in solving fractional differential equations is the Caputo fractional derivative. It allows to specify a quantity of integer order derivatives at the initial point. This quantity typically is available and can be measured. It is therefore not surprising that there is a vast literature dealing with fractional differential equations involving the Caputo fractional derivative [12–16]. The theory and application of fractional differential equations under both types of fractional derivatives have been discussed by many authors [11, 17–25]. Some potential applications have been studied in [26–28].

In the context of mathematical modelling, developing an accurate fractional differential equation is not a simple task. It requires an understanding of real physical phenomena involved. The real physical phenomena, however, are always pervaded with uncertainty. This is obvious when dealing with “living” materials such as soil, water, and microbial populations [29]. When a real physical phenomenon is modelled by a fractional differential equation, namely,

(1) $$\begin{array}{c} D_{a}^{\beta }x\left(t\right)=f\left(t,x\left(t\right)\right),\;\;\;0<\beta \leq 1,\;\;t>a,\\ x\left(t_{0}\right)=x_{0}, \end{array}$$

we cannot usually be sure that the model is perfect. For example, the initial value in (1) may not be known precisely. It may take any value in the form of “less than *x*_0_,” “about *x*_0_,” or “more than *x*_0_.” Classical mathematics, however, fail to cope with this situation. Therefore, it is necessary to have other theories in order to handle this issue. Various theories exist for describing this situation and the most popular one is the fuzzy set theory [30].

In order to obtain a more realistic model than (1), Agarwal et al. [31] have taken an initiative to introduce the concept of solution for fuzzy fractional differential equations. This contribution has motivated several authors to establish some results on the existence and uniqueness of solution (see [32]). In [33], the authors derived the explicit solution of fuzzy fractional differential equations using the Riemann-Liouville H-derivative. Recently, Salahshour et al. [34] applied fuzzy Laplace transforms [35] to solve fuzzy fractional differential equations. Basically, the proposed ideas are a generalisation of the theory and solution of fuzzy differential equations [36–41]. However, the authors considered fuzzy fractional differential equations under the Riemann-Liouville H-derivative. Again, it requires a quantity of fractional H-derivative of an unknown solution at the fuzzy initial point. In this paper, we propose a new interpretation of fuzzy fractional differential equations and present their solutions analytically and numerically. The proposed idea is a generalisation of the interpretation given in [42–49], where the authors used Zadeh's extension principle to interpret fuzzy differential equations. According to Mizukoshi et al. [43], this interpretation requires neither the concept of derivatives of a fuzzy function nor the use of selection theory to obtain a solution to the fuzzy differential equation.

This paper is organised in the following sequence. In Section 2, we recall some basic definitions and theoretical backgrounds needed in this paper. In Section 3, we present the solution of fuzzy fractional differential equations analytically and numerically. In Section 4, some numerical examples are given. Finally in Section 5, we give conclusions.

## 2. Basic Concepts

In this section, we briefly elaborate some definitions and important concepts of fractional calculus and fuzzy set. 

### 2.1. The Fractional Integral and Fractional Derivative

The following definitions of fractional integral and fractional derivative are adopted from [11].

**Definition 1**

A real function *x*(*t*), *t* > 0, is said to be in the space *C*_*μ*_, *μ* ∈ ℝ, if there exists a real number *p* > *μ*, such that *x*(*t*) = *t* ^*p*^ *x*_1_(*t*), where *x*_1_(*t*) ∈ *C*(0, *∞*) and it is said to be in the space *C*_*μ*_^*n*^ if and only if *x* ^*n*^ ∈ *C*_*μ*_, *n* ∈ *ℕ*.

**Definition 2**

The Riemann-Liouville fractional integral of *x* of order *β* > 0 with *a* ≥ 0 is defined as

(2) $$\begin{array}{c} I_{a}^{\beta }x\left(t\right)=\frac{1}{\Gamma \left(\beta \right)}\int_{a}^{t}{\left(t-s\right)}^{\beta -1}x\left(s\right)ds,\;t>a, \end{array}$$

and for *β* = 0, the Riemann-Liouville fractional integral of *x* is defined as

(3) $$\begin{array}{c} I_{a}^{0}x\left(t\right)=x\left(t\right). \end{array}$$

Here, Γ(*β*) is the well-known gamma function defined as

(4) $$\begin{array}{c} \Gamma \left(\beta \right)=\int_{0}^{\infty }t^{\beta -1}e^{-t}dt. \end{array}$$

**Definition 3**

The Caputo fractional derivative of *x* of order *β* > 0 with *a* ≥ 0 is defined as

(5) $$\begin{array}{c} {\;}^{c}D_{a}^{\beta }x\left(t\right)=\frac{1}{\Gamma \left(n-\beta \right)}\int_{a}^{t}{\left(t-s\right)}^{n-\beta -1}x^{\left(n\right)}\left(s\right)ds \end{array}$$

for *n* − 1 < *β* ≤ *n*, *n* ∈ *ℕ*, *t* ≥ *a*, *x* ∈ *C*_−1_^*n*^.

The following are two basic properties of the Caputo fractional derivative [50].

(1) Let *x* ∈ *C*_−1_^*n*^, *n* ∈ *ℕ*. Then ^*c*^ *D*_*a*_^*β*^ *x*, 0 ≤ *β* ≤ *n*, is well defined and ^*c*^ *D*_*a*_^*β*^ *x* ∈ *C*_−1_.
(2) Let *n* − 1 < *β* ≤ *n*, *n* ∈ *ℕ*, and *x* ∈ *C*_*μ*_^*n*^, *μ* ≥ −1. Then
  (6) $$\begin{array}{c} I_{a}^{\beta }\left({\;}^{c}D_{a}^{\beta }\right)x\left(t\right)=x\left(t\right)-\sum_{k=0}^{n-1}x^{\left(k\right)}\left(a\right)\frac{{\left(t-a\right)}^{k}}{k!}. \end{array}$$

The Laplace transform of the Caputo fractional derivative is given by [51]

(7) ℒ{ cDβx(t)} =sβX(s)−∑k=0n−1sβ−k−1x(k)(0); n−1<β≤n.

### 2.2. Fuzzy Set Theory

 According to Zadeh [30], a fuzzy set is a generalisation of a classical set that allows a membership function to take any value in the unit interval [0,1].

**Definition 4 (see [30])**

Let *U* be a universal set. A fuzzy set *A* in *U* is defined by a membership function *A*(*x*) that maps every element in *U* to the unit interval [0,1].

**Definition 5 (see [38])**

Let *A* be a fuzzy set defined in ℝ. *A* is called a fuzzy number if

(i) *A* is normal: there exists *x*_0_ ∈ ℝ such that *A*(*x*_0_) = 1;
(ii) *A* is convex: for all *x*, *y* ∈ ℝ and 0 ≤ *λ* ≤ 1, it holds that
  (8) $$\begin{array}{c} A\left(\lambda x+\left(1-\lambda \right)y\right)\geq \min\left(A\left(x\right),A\left(y\right)\right); \end{array}$$
(iii) *A* is upper semicontinuous: for any *x*_0_ ∈ ℝ, it holds that
  (9) $$\begin{array}{c} A\left(x_{0}\right)\geq \underset{x\to x_{0}^{\pm }}{\lim}A\left(x\right); \end{array}$$
(iv) $\;{\left[A\right]}^{0}=\bar{\left\{x\in \mathbb{R}|A(x)>0\right\}}$ is a compact subset of ℝ.

**Definition 6 (see [38])**

Let *A* be a fuzzy number defined in ℝ. The *α*-cut of *A* is the crisp set [*A*]^*α*^ that contains all elements in ℝ such that the membership value of *A* is greater than or equal to *α*; that is,

(10) $$\begin{array}{c} {\left[A\right]}^{\alpha }=\left\{x\in U\;|\;A\left(x\right)\geq \alpha \right\},\;\alpha \in \left(0,1\right]. \end{array}$$

For a fuzzy number *A*, its *α*-cuts are closed intervals in ℝ and we denote them by

(11) $$\begin{array}{c} {\left[A\right]}^{\alpha }=\left[a_{1}^{\alpha },a_{2}^{\alpha }\right]. \end{array}$$

**Definition 7 (see [52])**

A fuzzy number *A* is called a triangular fuzzy number if its membership function has the following form:

(12) $$\begin{array}{c} A\left(x\right)=\{\begin{array}{cc} 0, & \text{if}\;\;x<a,\\ \frac{x-a}{b-a}, & \text{if}\;\;a\leq x<b,\\ \frac{c-x}{c-b}, & \text{if}\;\;b\leq x\leq c,\\ 0, & \text{if}\;\;x>c, \end{array} \end{array}$$

and its *α*-cuts are simply [*A*]^*α*^ = [*a* + *α*(*b* − *a*), *c* − *α*(*c* − *b*)], *α* ∈ (0,1].

In this paper, we denote *A* = (*a*, *b*, *c*) as the triangular fuzzy number and *ℱ*(ℝ) as the set of all triangular fuzzy numbers.

Any crisp function can be extended to take fuzzy set as arguments by applying Zadeh's extension principle [30]. Let *f* be a function from *X* to *Y*. Given a fuzzy set *A* in *X*, we want to find a fuzzy set *B* = *f*(*A*) in *Y* that is induced by *f*. If *f* is a strictly monotone, then we can extend *f* to fuzzy set as follows:

(13) $$\begin{array}{c} f\left(A\right)\left(y\right)=\{\begin{array}{cc} A\left(f^{-1}\left(y\right)\right), & \text{if}\;\;y\in \text{range}\left(f\right),\\ 0, & \text{if}\;\;y\notin \text{range}\left(f\right). \end{array} \end{array}$$

It is clear that (13) can be easily calculated by determining the membership at the endpoints of the *α*-cuts of *A*. However, in general, the process of finding the fuzzy set *B* = *f*(*A*) is more complicated and cannot be gathered easily. For example, if *f* is nonmonotone, then the problem can arise when two or more distinct points in *X* are mapped to the same point in *Y*. If this is the case, then the above equation may take two or more different values. This requires a new extension of (13) as shown below:

(14) $$\begin{array}{c} f\left(A\right)\left(y\right)=\{\begin{array}{cc} \underset{x\in f^{-1}\left(y\right)}{\sup}A\left(x\right), & \text{if}\;\;y\in \text{range}\left(f\right),\\ 0, & \text{if}\;\;y\notin \text{range}\left(f\right), \end{array} \end{array}$$

where

(15) $$\begin{array}{c} f^{-1}\left(y\right)=\left\{x\in X\;|\;f\left(x\right)=y\right\}. \end{array}$$

Some computational methods to compute (14) can be found in [53, 54].

**Theorem 8 (see [55])**

If *f* : ℝ → ℝ is continuous, then *f* : *ℱ*(ℝ) → *ℱ*(ℝ) is well defined and

(16) $$\begin{array}{c} {\left[f(A)\right]}^{\alpha }=f\left({\left[A\right]}^{\alpha }\right),\;\;\forall \alpha \in \left[0,1\right],\;\;\forall A\in ℱ\left(\mathbb{R}\right), \end{array}$$

where *f*(*A*) = {*f*(*u*) | *u* ∈ [*A*]^*α*^}.

For *A*, *B* ∈ *ℱ*(ℝ) and *λ* ∈ ℝ, the sum *A* + *B* and the product *λA* are defined as follows, respectively:

(17) $$\begin{array}{c} {\left[A+B\right]}^{\alpha }={\left[A\right]}^{\alpha }+{\left[B\right]}^{\alpha },\\ {\left[\lambda B\right]}^{\alpha }=\lambda {\left[A\right]}^{\alpha } \end{array}$$

for each *α* ∈ [0,1].

**Definition 9 (see [56])**

If *A* and *B* are two fuzzy numbers, then the distance *D* between *A* and *B* is defined as

(18) $$\begin{array}{c} D\left(A,B\right)=\underset{\alpha \in [0,1]}{\sup}\max\left\{\left|a_{1}^{\alpha }-b_{1}^{\alpha }\right|,\left|a_{2}^{\alpha }-b_{2}^{\alpha }\right|\right\}. \end{array}$$

In [57], the authors have shown that (*ℱ*(ℝ), *D*) is a complete metric space and the following properties are well known:

- *D*(*A* + *C*, *B* + *C*) = *D*(*A*, *B*), ∀*A*, *B*, *C* ∈ *ℱ*(ℝ),
- *D*(*λA*, *λB*) = |*λ* | *D*(*A*, *B*), ∀*A*, *B* ∈ *ℱ*(ℝ) and *λ* ∈ ℝ,
- *D*(*A* + *B*, *C* + *D*) ≤ *D*(*A*, *C*) + *D*(*B*, *D*), ∀*A*, *B*, *C*, *D* ∈ *ℱ*(ℝ).

## 3. Fuzzy Fractional Differential Equations

In this section, we present analytical and numerical solutions of fuzzy fractional differential equations.

### 3.1. Analytical Solution of Fuzzy Fractional Differential Equations

First, let us consider the following fractional differential equation:

(19) $$\begin{array}{c} {\;}^{c}D_{a}^{\beta }x\left(t\right)=f\left(t,x\left(t\right)\right),\\ x\left(t_{0}\right)=x_{0}, \end{array}$$

where *f* : [*t*_0_, *T*] × ℝ → ℝ is a real-valued function, *x*_0_ ∈ ℝ, and *β* ∈ (0,1]. If *β* = 1, then (19) becomes an ordinary differential equation.

Assume that the initial value is replaced by a fuzzy number; then we have the following fuzzy fractional differential equation:

(20) $$\begin{array}{c} {\;}^{c}D_{a}^{\beta }X\left(t\right)=f\left(t,X\left(t\right)\right),\\ X\left(t_{0}\right)=X_{0}, \end{array}$$

where *X*_0_ ∈ *ℱ*(ℝ) and *β* ∈ (0,1]. If *β* = 1, then (20) becomes a fuzzy differential equation.

In order to find the solution of (20), we first find the solution of (19). By taking the Laplace transform on both sides of (19), we get

(21) $$\begin{array}{c} ℒ\left[{\;}^{c}D_{a}^{\beta }x\left(t\right)\right]=ℒ\left[f\left(t,x\left(t\right)\right)\right]. \end{array}$$

It follows that

(22) $$\begin{array}{c} s^{\beta }ℒ\left\{x\left(t\right)\right\}-x\left(t_{0}\right)s^{\beta -1}=ℒ\left[f\left(t,x\left(t\right)\right)\right]. \end{array}$$

Assume that after simplifying (22), we get

(23) $$\begin{array}{c} ℒ\left[x\left(t\right)\right]=m\left(s\right). \end{array}$$

Then by taking the inverse Laplace transform to (23), we have

(24) $$\begin{array}{c} x\left(t\right)={ℒ}^{-1}\left[m\left(s\right)\right]=g\left(t,\beta ,x_{0}\right), \end{array}$$

for *t* ∈ [*t*_0_, *T*] and *x*_0_ ∈ ℝ. In order to find the solution of (20), we fuzzify (24) using Zadeh's extension principle. Hence, we have

(25) $$\begin{array}{c} X\left(t\right)=\hat{g}\left(t,\beta ,X_{0}\right), \end{array}$$

which is the solution of (20).

This procedure can be shown precisely in the following theorem.

**Theorem 10**

Let *G* be an open set in ℝ and [*X*_0_]^*α*^ ∈ *ℱ*(ℝ) ⊂ *G*. Suppose that *f* is continuous and that for each *β* ∈ (0,1] and each *x*_0_ ∈ *G* there exists a unique solution *g*(·, *β*, *x*_0_) of the problem (19) and that *g*(*t*, *β*, ·) is continuous in *G* for each *t* ∈ [*t*_0_, *T*] fixed. Then, there exists a unique fuzzy solution $X(t)=\hat{g}(t,\beta ,X_{0})$ of the problem (20).

**Proof**

The proof of this theorem uses the basic idea found in [46]. Since *f* is continuous, there exists a unique solution *g*(*t*, *β*, *x*_0_). This solution is well defined and continuous in *G*, for each *t* ∈ [*t*_0_, *T*] fixed. Then, from Theorem 8, we have $\hat{g}(t,\beta ,\cdot ):ℱ(G)\to ℱ(\mathbb{R})$, which is continuous and well defined. Therefore, there exists a unique fuzzy solution of the form $X(t)=\hat{g}(t,\beta ,X_{0})$ for the problem (20).

**Remark 11**

The existence of *X*(*t*) is guaranteed by Theorem 8 since *x*(*t*) is continuous.

Moreover, in order to have valid level sets, *X*(*t*) should satisfy the following Staking Theorem [58].

**Theorem 12**

If *X* : [*t*_0_, *T*] → *ℱ*(ℝ) is a fuzzy solution of (20) and denoting [*X*(*t*)]^*α*^ = [*x*_1_^*α*^(*t*), *x*_2_^*α*^(*t*)] for *α* ∈ [0,1], then

- [*X*(*t*)]^*α*^ is nonempty compact subset of ℝ;
- [*X*(*t*)]^*α*_2_^⊆[*X*(*t*)]^*α*_1_^ for 0 ≤ *α*_1_ ≤ *α*_2_ ≤ 1;
- [*X*(*t*)]^*α*^ = ⋂_*n*=1_^*∞*^[*X*(*t*)]^*α*_*n*_^ for any nondecreasing sequence *α*_*n*_ → *α* in [0,1].

In the following result, we will show that *x*_1_^*α*^(*t*) and *x*_2_^*α*^(*t*) do not interchange at all *t* ∈ [*t*_0_, *∞*).

**Theorem 13**

If *X*(*t*) = *g*(*t*, *β*, *X*_0_) is obtained by using Theorem 10 and [*X*(*t*)]^*α*^ = [*x*_1_^*α*^(*t*), *x*_2_^*α*^(*t*)] for *α* ∈ [0,1], then *x*_1_^*α*^(*t*) and *x*_2_^*α*^(*t*) do not interchange at all *t* ∈ [*t*_0_, *∞*).

**Proof**

We know that *X*(*t*) is obtained by Zadeh's extension principle through Theorem 10; then its membership function has the following form:

(26) $$\begin{array}{c} X\left(t\right)\left(y\right)=\{\begin{array}{cc} \underset{x\in g^{-1}(t,\beta ,y)}{\sup}X_{0}\left(x\right), & \text{if}\;\;\;y\in \text{range}\left(g\right),\\ 0, & \text{if}\;\;y\notin \text{range}\left(g\right). \end{array} \end{array}$$

It follows that

(27) $$\begin{array}{c} x_{1}^{\alpha }\left(t\right)=\min\left\{g\left(t,\beta ,u\right)\;|\;u\in \left[x_{0,1}^{\alpha },x_{0,2}^{\alpha }\right]\right\},\\ x_{2}^{\alpha }\left(t\right)=\max\left\{g\left(t,\beta ,u\right)\;|\;u\in \left[x_{0,1}^{\alpha },x_{0,2}^{\alpha }\right]\right\} \end{array}$$

for *α* ∈ [0,1]. It is obvious that

(28) $$\begin{array}{c} x_{1}^{\alpha }\left(t\right)\leq x_{2}^{\alpha }\left(t\right). \end{array}$$

This holds for all *t* ∈ [*t*_0_, *∞*). This completes the proof.

In general, the solution of (20) may not be found analytically. Therefore, a numerical method must be proposed.

### 3.2. Numerical Solution of Fuzzy Fractional Differential Equations

Fractional Euler method under the Caputo fractional derivative has been proposed in [59]. However, in order to approximate the solution of fuzzy fractional differential equations, the fractional Euler method has to be extended in the fuzzy setting. In this case, Zadeh's extension principle plays an important role.

Let *x*(*t*) be the solution of (19). The first two terms of fractional Taylor series for *x*(*t*) at *t*_*i*_ can be written as [59]

(29) $$\begin{array}{c} x\left(t_{i+1}\right)\approx x\left(t_{i}\right)+{}^{c}D^{\beta }x\left(t_{i}\right)\frac{h^{\beta }}{\Gamma \left(\beta +1\right)}. \end{array}$$

From (19), we have

(30) $$\begin{array}{c} x\left(t_{i+1}\right)\approx x\left(t_{i}\right)+\frac{h^{\beta }}{\Gamma \left(\beta +1\right)}f\left(t_{i},x\left(t_{i}\right)\right). \end{array}$$

Let *w*_*i*+1_ ≈ *x*(*t*_*i*+1_); then we have the following fractional Euler method [59]:

(31) $$\begin{array}{c} w_{i+1}=w_{i}+\frac{h^{\beta }}{\Gamma \left(\beta +1\right)}f\left(t_{i},w_{i}\right), \end{array}$$

for *i* = 0,1, 2,…, *N*. Let *g*(*β*, *h*, *t*_*i*_, *w*_*i*_) = *w*_*i*_ + (*h* ^*β*^/Γ(*β* + 1))*f*(*t*_*i*_, *w*_*i*_); then (31) becomes

(32) $$\begin{array}{c} w_{i+1}=g\left(\beta ,h,t_{i},w_{i}\right). \end{array}$$

To approximate the solution of (20), (32) will be fuzzified using Zadeh's extension principle. We then obtain the following fuzzy fractional Euler method:

(33) $$\begin{array}{c} W_{i+1}=g\left(\beta ,h,t_{i},W_{i}\right) \end{array}$$

for *i* = 0,1,…, *N*, and *W*_*i*_ ∈ *ℱ*(ℝ). Please note that if *β* = 1, then this fuzzy fractional Euler method becomes fuzzy Euler method as proposed in [47]. The membership function of *g* in (33) can be defined as

(34) g(β,h,ti,Wi)(y) ={sup⁡x∈g−1(β,h,ti,y)⁡Wi(x),if  y∈range(g),0,if  y∉range(g).

In general, the computation of (34) is not an easy task. By using the concept of *α*-cuts, (34) can be calculated as follows:

(35) $$\begin{array}{c} w_{i+1,1}^{\alpha }=\min\left\{\left(u+\frac{h^{\beta }}{\Gamma \left(\beta +1\right)}f\left(t_{i},u\right)\right)\;|\;u\in \left[w_{i,1}^{\alpha },w_{i,2}^{\alpha }\right]\right\},\\ w_{i+1,2}^{\alpha }=\max\left\{\left(u+\frac{h^{\beta }}{\Gamma \left(\beta +1\right)}f\left(t_{i},u\right)\right)\;|\;u\in \left[w_{i,1}^{\alpha },w_{i,2}^{\alpha }\right]\right\}. \end{array}$$

The optimisation problems in (35) are performed as follows.

- If *g*(*β*, *h*, *t*_*i*_, *u*) is increasing or decreasing on the interval [*w*_*i*,1_^*α*^, *w*_*i*,2_^*α*^], then the optimal solutions are obtained at the endpoints of that interval (see Algorithm 1).
- If *g*(*β*, *h*, *t*_*i*_, *u*) is nonmonotone, we first split the interval [*w*_*i*,1_^*α*^, *w*_*i*,2_^*α*^] into several subintervals and then solve the optimisation problems on the subintervals. By taking the minimum and maximum of all the results, we obtain the optimal solutions on the interval [*w*_*i*,1_^*α*^, *w*_*i*,2_^*α*^]. These procedures are given in Algorithm 2.

## 4. Numerical Examples

In this section, we present three examples for solving fuzzy fractional differential equations.

**Example 1**

Consider the following linear fuzzy fractional differential equation:

(36) $$\begin{array}{c} {\;}^{c}D_{0}^{\beta }X\left(t\right)=X\left(t\right),\\ X\left(0\right)=X_{0}, \end{array}$$

where *β* ∈ (0,1], *t* > 0, and *X*_0_ is any triangular fuzzy number.

This problem is a generalisation of the following fractional differential equation:

(37) $$\begin{array}{c} {\;}^{c}D_{0}^{\beta }x\left(t\right)=x\left(t\right),\\ x\left(t\right)=x_{0}, \end{array}$$

where *β* ∈ (0,1], *t* > 0, and *x*_0_ is a real number.

In order to find the solution of (36), we first find the solution of (37). By taking the Laplace transform on both sides of (37), we have

(38) $$\begin{array}{c} ℒ\left[{\;}^{c}D_{0}^{\beta }x\left(t\right)\right]=ℒ\left[x\left(t\right)\right]. \end{array}$$

We then obtain

(39) $$\begin{array}{c} s^{\beta }ℒ\left\{x\left(t\right)\right\}-x\left(t_{0}\right)s^{\beta -1}=ℒ\left\{x\left(t\right)\right\}. \end{array}$$

Simplifying (39), we get

(40) $$\begin{array}{c} ℒ\left\{x\left(t\right)\right\}=\frac{x_{0}s^{\beta -1}}{s^{\beta }-1}. \end{array}$$

By taking the inverse Laplace transform to (40), we obtain

(41) $$\begin{array}{c} x\left(t\right)=x_{0}{ℒ}^{-1}\left\{\frac{s^{\beta -1}}{s^{\beta }-1}\right\}, \end{array}$$

which finally has the following solution:

(42) $$\begin{array}{c} x\left(t\right)=x_{0}E_{\beta }\left(t^{\beta }\right), \end{array}$$

where *E*_*β*_(∗) is the Mittag-Leffler function defined as

(43) $$\begin{array}{c} E_{\beta }\left(z\right)=\sum_{k=0}^{\infty }\frac{z^{k}}{\Gamma \left(\beta k+1\right)},\;\beta >0. \end{array}$$

By using Zadeh's extension principle to (42) in relation to *x*_0_, we obtain

(44) $$\begin{array}{c} X\left(t\right)=X_{0}E_{\beta }\left(t^{\beta }\right), \end{array}$$

which is the solution of (36).

Let [*X*(*t*)]^*α*^ = [*x*_1_^*α*^(*t*), *x*_2_^*α*^(*t*)] and *X*_0_ = (1,2, 3), where [*X*_0_]^*α*^ = [1 + *α*, 3 − *α*] for *α* ∈ [0,1]; then the first ten terms of (44) can be expressed as follows:

(45) x1α(t)=(1+α)(1+tβΓ(β+1)+t2βΓ(2β+1)     +t3βΓ(3β+1)+t4βΓ(4β+1)     +t5βΓ(5β+1)+t6βΓ(6β+1)     +t7βΓ(7β+1)+t8βΓ(8β+1)     +t9βΓ(9β+1)),x2α(t)=(3−α)(1+tβΓ(β+1)+t2βΓ(2β+1)     +t3βΓ(3β+1)+t4βΓ(4β+1)     +t5βΓ(5β+1)t6βΓ(6β+1)+t7βΓ(7β+1)     +t8βΓ(8β+1)+t9βΓ(9β+1)).

**Figure 1:**
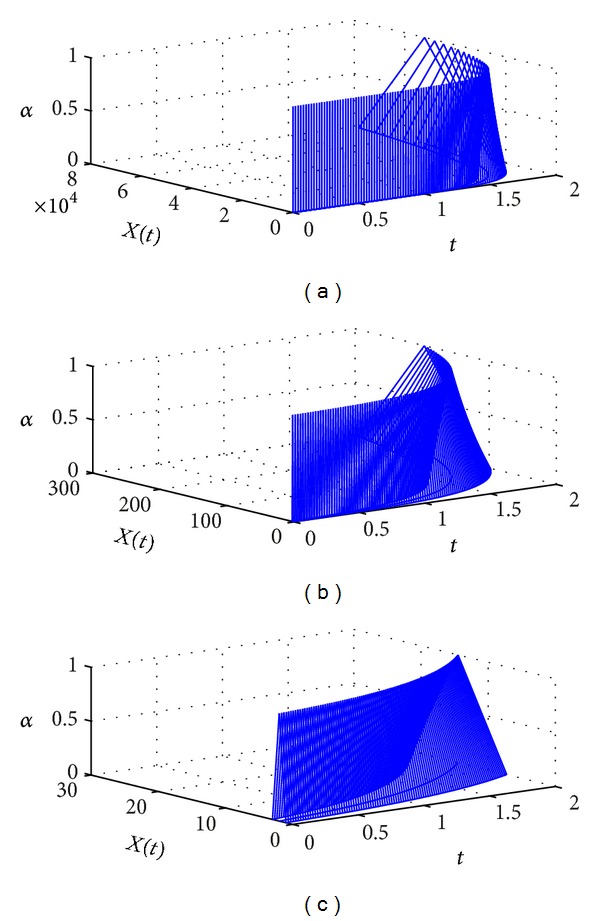
The numerical solution of (36) for (a) *β* = 0.6, (b) *β* = 0.8, and (c) *β* = 1.

Clearly, (45) are the valid *α*-cuts of the solution of (36). For numerical approximation, we set *t* ∈ [0,2] and *N* = 100. By using Algorithm 1, the results for different values of *β* are plotted in Figure 1. From the graphs, we can see that if *β* approaches 1, the approximate solutions will approach the approximate solution of fuzzy differential equation. Numerical values at *t* = 2 for different values of *β* are listed in Table 1.

**Example 2**

Consider the following linear fuzzy fractional differential equation:

(46) $$\begin{array}{c} {\;}^{c}D_{0}^{\beta }X\left(t\right)=-X\left(t\right),\\ X\left(0\right)=X_{0}. \end{array}$$

The nonfuzzy problem associated with (46) is

(47) $$\begin{array}{c} {\;}^{c}D_{0}^{\beta }x\left(t\right)=-x\left(t\right),\\ x\left(0\right)=x_{0}. \end{array}$$

In order to find the solution of (46), we first find the solution of (47). By taking the Laplace transform on both sides of (47), we have

(48) $$\begin{array}{c} ℒ\left\{{\;}^{c}D_{0}^{\beta }x\left(t\right)\right\}=-ℒ\left\{x\left(t\right)\right\}. \end{array}$$

It follows that

(49) $$\begin{array}{c} s^{\beta }ℒ\left\{x\left(t\right)\right\}-x\left(t_{0}\right)s^{\beta -1}=-ℒ\left\{x\left(t\right)\right\}. \end{array}$$

After simplifying, we get

(50) $$\begin{array}{c} ℒ\left\{x\left(t\right)\right\}=\frac{x_{0}s^{\beta -1}}{s^{\beta }+1}. \end{array}$$

By taking the inverse Laplace transform to (50), we obtain

(51) $$\begin{array}{c} x\left(t\right)=x_{0}{ℒ}^{-1}\left\{\frac{s^{\beta -1}}{s^{\beta }+1}\right\}, \end{array}$$

which finally has the following solution:

(52) $$\begin{array}{c} x\left(t\right)=x_{0}E_{\beta }\left(-t^{\beta }\right), \end{array}$$

where *E*_*β*_(∗) is the Mittag-Leffler function. Using Zadeh's extension principle to (52) in relation to *x*_0_, we obtain the solution of (46) as follows:

(53) $$\begin{array}{c} X\left(t\right)=X_{0}E_{\beta }\left(-t^{\beta }\right). \end{array}$$

Let [*X*(*t*)]^*α*^ = [*x*_1_^*α*^(*t*), *x*_2_^*α*^(*t*)] and *X*_0_ = (2,3, 4) where [*X*_0_]^*α*^ = [*α* + 2,4 − *α*] for *α* ∈ [0,1]; then the first ten terms of (52) can be expressed as follows:

(54) x1α(t)=(α+2)(−1−tβΓ(β+1)−t2βΓ(2β+1)     −t3βΓ(3β+1)−t4βΓ(4β+1)−t5βΓ(5β+1)     −t6βΓ(6β+1)−t7βΓ(7β+1)     −t8βΓ(8β+1)−t9βΓ(9β+1)),x2α(t)=(4−α)(−1−tβΓ(β+1)−t2βΓ(2β+1)     −t3βΓ(3β+1)−t4βΓ(4β+1)−t5βΓ(5β+1)     −t6βΓ(6β+1)−t7βΓ(7β+1)     −t8βΓ(8β+1)  −t9βΓ(9β+1)).

**Figure 2:**
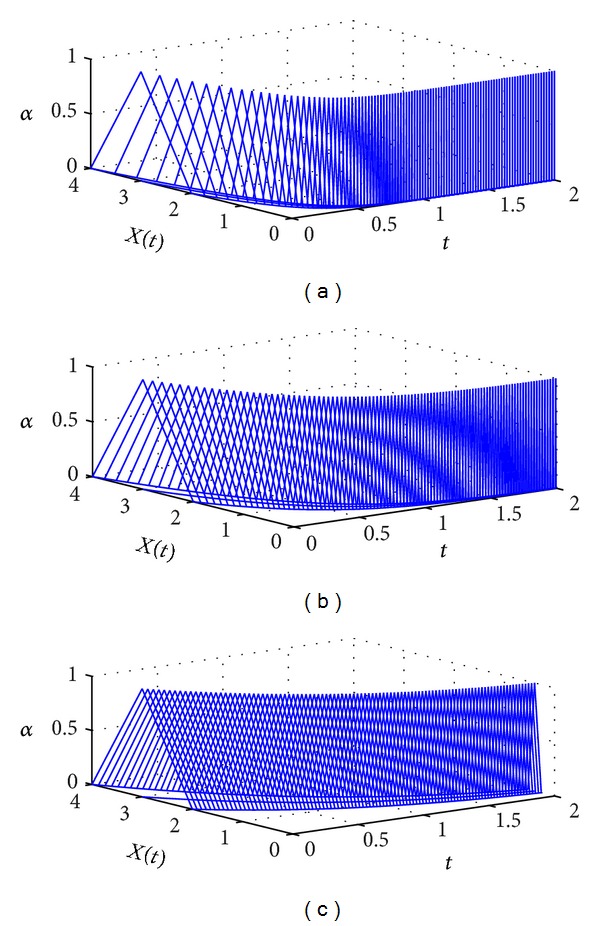
The numerical solutions of (46) for (a) *β* = 0.6, (b) *β* = 0.8, and (c) *β* = 1.

Clearly, (54) are the valid *α*-cuts of the solution of (46). By using Algorithm 1 with the same interval *t* and interval *N* as in Example 1, the numerical solutions of (46) for different values of *β* are plotted in Figure 2. Again, we can see that the numerical solutions will approach the numerical solution of fuzzy differential equation as *β* increases to 1. Numerical solutions at *t* = 2 for different values of *β* are listed in Table 2.

In the following example, we provide detailed procedures for solving a nonlinear fuzzy fractional differential equation.

**Example 3**

Let us consider the following problem:

(55) $$\begin{array}{c} {\;}^{c}D_{0}^{\beta }X\left(t\right)=\cos\left(tX\right),\\ X\left(0\right)=X_{0}, \end{array}$$

where *β* ∈ (0,1], *t* = [0,5], and *X*_0_ is any triangular fuzzy number.

To solve this problem, we use Algorithm 2. First, let [*X*_0_]^*α*^ = [*x*_0,1_^*α*^, *x*_0,2_^*α*^]. We discretise *α* up to 11 points, which are *α*_0_ = 0 < *α*_1_ = 0.1 < ⋯<*α*_10_ = 1. Let *X*_0_ = *W*_0_; then we have

(56) w1,1α10=g(β,h,t0,w0,1α10)=g(β,h,t0,w0,2α10)=w1,2α10,w2,1α9=min⁡[min⁡u∈[w1,1α9,w1,1α10]g(β,h,t1,u),w1,1α10,min⁡u∈[w1,2α10,w1,2α9]g(β,h,t1,u)],w2,2α9=max⁡[max⁡u∈[w1,1α9,w1,1α10]g(β,h,t1,u),w1,2α10,max⁡u∈[w1,2α10,w1,2α9]g(β,h,t1,u)],                        ⋮wN+1,1α0=min⁡[min⁡u∈[wN,1α0,wN,1α1]g(β,h,tN,u),wN,1α1,min⁡u∈[wN,2α1,wN,2α0]g(β,h,tN,u)],wN+1,2α0=max⁡[max⁡u∈[wN,1α0,wN,1α1]g(β,h,tN,u),wN,2α1,max⁡u∈[wN,2α1,wN,2α0]g(β,h,tN,u)],

where

(57) $$\begin{array}{c} g\left(\beta ,h,t_{i},u\right)=u+\frac{h^{\beta }}{\Gamma \left(\beta +1\right)}\cos\left(t_{i}u\right),\;u\in \left[w_{i,1}^{\alpha_{j}},w_{i,2}^{\alpha_{j}}\right] \end{array}$$

for *i* = 0,1,…, *N* and *j* = 0,1,…, 10.

**Figure 3:**
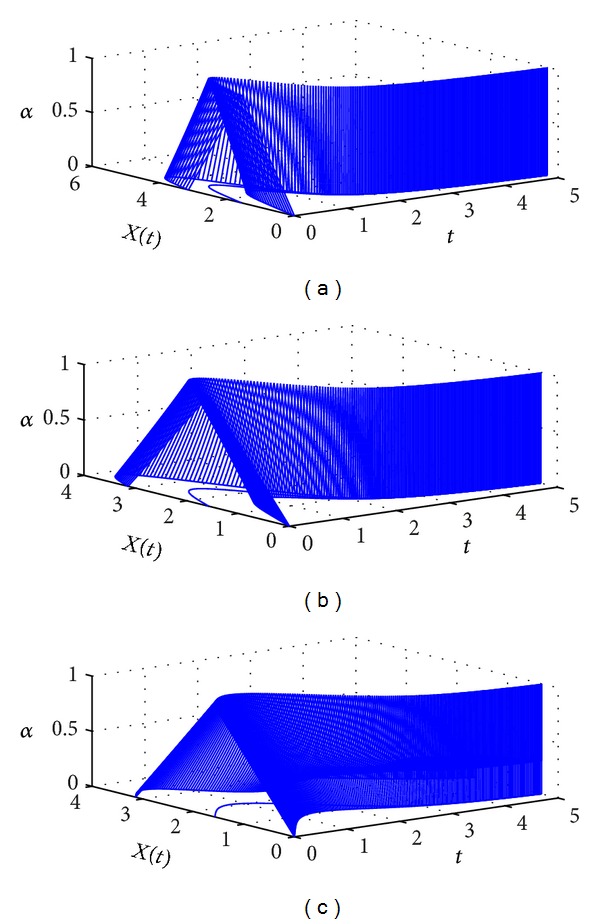
The approximate solutions of (55) for (a) *β* = 0.6, (b) *β* = 0.8, and (c) *β* = 1.

Let *X*_0_ = (0, *π*/2, *π*) and *N* = 200; then these procedures will result in the approximate solutions of (55) at different values of *β* as plotted in Figure 3. From the graphs, we can see that the numerical solutions approach to the numerical solution of fuzzy differential equation as *β* approaches 1. Numerical solutions at *t* = 5 at different values of *β* are listed in Table 3.

## 5. Conclusions

 In this paper, we have studied a fuzzy fractional differential equation and presented its solution using Zadeh's extension principle. The classical fractional Euler method has also been extended in the fuzzy setting in order to approximate the solutions of linear and nonlinear fuzzy fractional differential equations. Final results showed that the solution of fuzzy fractional differential equations approaches the solution of fuzzy differential equations as the fractional order approaches the integer order.

## Figures and Tables

**Algorithm 1:**
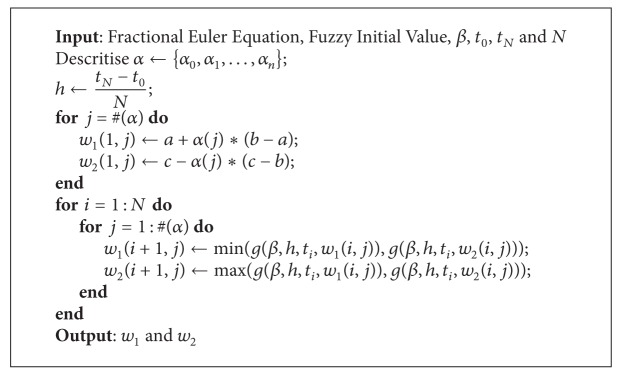
Fuzzy fractional Euler method for a linear problem.

**Algorithm 2:**
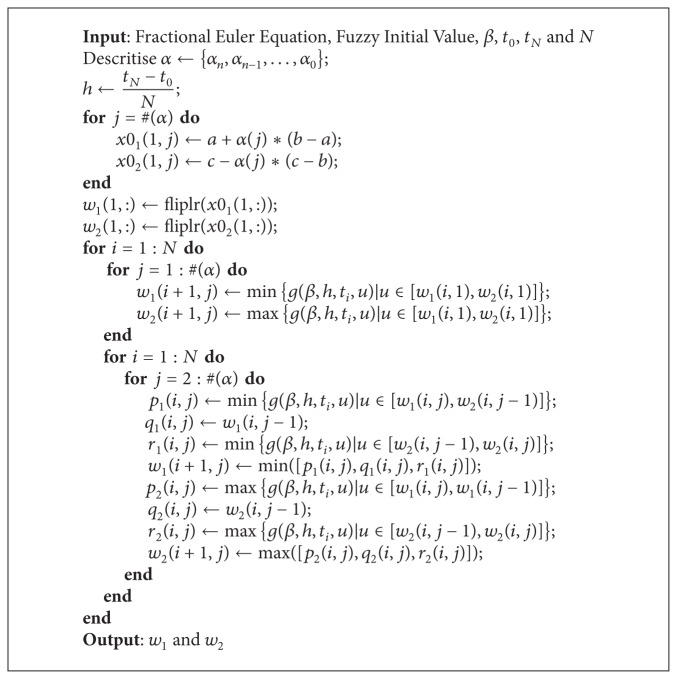
Fuzzy fractional Euler method for a nonlinear problem.

**Table 1:** Numerical solutions of Example 1 with different values of *β*.

*α*	*β* = 0.6		*β* = 0.8		*β* = 1	
	*x*_1_^*β*^(*t*)	*x*_2_^*β*^(*t*)	*x*_1_^*β*^(*t*)	*x*_2_^*β*^(*t*)	*x*_1_^*β*^(*t*)	*x*_2_^*β*^(*t*)
0.0	26063.941926	78191.825779	98.369851	295.109553	7.244646	21.733938
0.1	28670.336119	75585.431586	108.206836	285.272568	7.969110	21.009473
0.2	31276.730311	72979.037394	118.043821	275.435583	8.693575	20.285009
0.3	33883.124504	70372.643201	127.880806	265.598598	9.418039	19.560544
0.4	36489.518697	67766.249008	137.717791	255.761613	10.142504	18.836079
0.5	39095.912889	65159.854816	147.554776	245.924627	10.866969	18.111615
0.6	41702.307082	62553.460623	157.391761	236.087642	11.591433	17.387150
0.7	44308.701274	59947.066430	167.228746	226.250657	12.315898	16.662686
0.8	46915.095467	57340.672238	177.065732	216.413672	13.040363	15.938221
0.9	49521.489660	54734.278045	186.902717	206.576687	13.764827	15.213756
1.0	52127.883852	52127.883852	196.739702	196.739702	14.489292	14.489292

**Table 2:** Numerical solutions of Example 2 with different values of *β*.

*α*	*β* = 0.6		*β* = 0.8		*β* = 1	
	*x*_1_^*β*^(*t*)	*x*_2_^*β*^(*t*)	*x*_1_^*β*^(*t*)	*x*_2_^*β*^(*t*)	*x*_1_^*β*^(*t*)	*x*_2_^*β*^(*t*)
0.0	0.000024	0.000048	0.016304	0.032608	0.265239	0.530478
0.1	0.000025	0.000047	0.017119	0.031793	0.278501	0.517216
0.2	0.000026	0.000046	0.017934	0.030978	0.291763	0.503954
0.3	0.000027	0.000044	0.018750	0.030163	0.305024	0.490692
0.4	0.000029	0.000043	0.019565	0.029347	0.318286	0.477430
0.5	0.000030	0.000042	0.020380	0.028532	0.331548	0.464168
0.6	0.000031	0.000041	0.021195	0.027717	0.344810	0.450906
0.7	0.000032	0.000040	0.022010	0.026902	0.358072	0.437644
0.8	0.000033	0.000038	0.022826	0.026087	0.371334	0.424382
0.9	0.000035	0.000037	0.023641	0.025271	0.384596	0.411120
1.0	0.000036	0.000036	0.024456	0.024456	0.397858	0.397858

**Table 3:** Numerical solutions of Example 3 with different values of *β*.

*α*	*β* = 0.6		*β* = 0.8		*β* = 1	
	*x*_1_^*β*^(*t*)	*x*_2_^*β*^(*t*)	*x*_1_^*β*^(*t*)	*x*_2_^*β*^(*t*)	*x*_1_^*β*^(*t*)	*x*_2_^*β*^(*t*)
0.0	0.316778	0.316778	0.320052	0.320052	0.328592	1.645658
0.1	0.316778	0.316778	0.320052	0.320052	0.328592	1.645476
0.2	0.316778	0.316778	0.320052	0.320052	0.328593	1.643608
0.3	0.316778	0.316778	0.320052	0.320052	0.328594	0.328664
0.4	0.316778	0.316778	0.320052	0.320052	0.328594	0.328629
0.5	0.316778	0.316778	0.320052	0.320052	0.328595	0.328617
0.6	0.316778	0.316778	0.320052	0.320052	0.328596	0.328610
0.7	0.316778	0.316778	0.320052	0.320052	0.328597	0.328606
0.8	0.316778	0.316778	0.320052	0.320052	0.328598	0.328604
0.9	0.316778	0.316778	0.320052	0.320052	0.328599	0.328602
1.0	0.316778	0.316778	0.320052	0.320052	0.328600	0.328600
